# Effects of air on the dosimetric robustness of treatment plans for prostate cancer in the presence of intrafractional, anatomical changes during online adaptive radiotherapy

**DOI:** 10.1002/acm2.70675

**Published:** 2026-07-07

**Authors:** Nika Guberina, Aymane Khouya, Alina Santiago Garcia, Christoph Pöttgen, Thomas Gauler, Gerrit Fischedick, Christopher Darr, Toke Printz Ringbæk, Maja Guberina, Martin Stuschke

**Affiliations:** ^1^ Department of Radiotherapy West German Cancer Center University Hospital Essen University of Duisburg Essen Essen Germany; ^2^ German Cancer Consortium (DKTK) Partner Site University Hospital Essen Essen Germany; ^3^ Department of Urology West German Cancer Center University Hospital Essen Essen Germany

**Keywords:** adaptive radiation therapy, dose inhomogeneity, plan robustness, prostate cancer

## Abstract

**Background:**

In the online adaptive platform, a linear Boltzmann‐transport‐equation‐solver (LBTE) as calculation algorithm is implemented for dose optimization. This class‐C‐optimizer is known to counteract the dose decrease at boundaries of small fields due to electronic disequilibrium by increasing the fluence to achieve the goals of dose homogeneity within the PTV.

**Purpose:**

To analyze the dosimetric sensitivity of adaptive treatment plans for prostate cancer to intrafractional anatomic changes in the presence of rectal air using a CBCT‐based adaptive platform.

**Methods:**

In the first part of the study planning CT scans (CT_plan_) from eight patients treated with an air‐filled endorectal balloon and two patients presenting larger rectal air pockets were included in a simulation study. The dataset was used to assess the sensitivity of LBTE‐optimized treatment plans to intrafractional density variations and to changes in the overlap between the planning target volume (PTV) and an air cavity (PTV∩Air structure). Density changes resulting from anatomical shifts were simulated using water overrides (WOR) in defined shift regions within the PTV∩Air structure. These shift regions were created by dorsally expanding the clinical target volume (CTV) by margins of 2 mm or 5 mm. Additionally, a WOR of the entire rectum was applied. For each WOR scenario, D_max_, D_1_ and D_1cc_ values were recorded within the fixed PTV∩Air structure and within the rectal wall, which was shifted according to the applied margin. A mixed linear model was built to describe dosimetric characteristics in dependence of the WOR scenario. Additionally, intrafractional variations of D_max_ within the PTV were assessed in 101 dose fractions from 15 patients who were treated with a rectal balloon and underwent online adaptive radiotherapy. All doses were normalized by the prescribed dose.

**Results:**

WOR within PTV∩Air‐structure overlap resulted in increases of the analyzed dosimetric characteristics. D_1cc_ increased with WOR within shift margins of 2 and 5 mm and within the entire rectal cavity by 2.2% ± 0.7%, 5.5% ± 0.7%, and 8.7% ± 1.0%, respectively (*p* < 0.0001, *F*‐test). The corresponding dose increases within the rectal wall were slightly smaller, with D_1cc_​ increases of 1.4% ± 0.6%, 1.7% ± 0.9%, and 5.0% ± 0.5%. Sensitivity to WOR in the PTV∩Air using a 5 mm shift margin was markedly lower when the same treatment plans were recalculated with AAA compared with LBTE (*p *= 0.0020, signed‐rank test). On average, AAA detected only 43% ± 12% of the WOR effect predicted by LBTE. For prostate cancer patients treated with a rectal balloon, D_max_, D_1_ and D_1cc_ changes in LBTE‐optimized plans showed 90% confidence intervals of [−0.05–0.05] due to intrafractional motion, suggesting that posterior tissue shifts of 5 mm or less do not have a clinically relevant impact.

**Conclusions:**

PTV∩Air larger than 5 mm led to LBTE‐optimized treatment plans, that are sensitive to anatomic soft tissue shifts larger than 5 mm in posterior direction with respect to dose homogeneity. PTV∩Air‐overlaps up to 5 mm led to LBTE‐optimized treatment plans, that showed only minor sensitivities to anatomic soft tissue shifts of 2–5 mm in posterior direction with respect to dose homogeneity. During online adaptive radiotherapy, the presence of rectal air pockets or an air‐filled rectal balloon did not result in clinically relevant dose increases, provided that intrafractional motion–induced soft tissue shifts remained within 5 mm in the posterior direction.

## 1 INTRODUCTION

Precision of radiation therapy for tumors in the pelvic region can be enhanced through online adaptive radiotherapy (ART), as this approach accounts for the high organ mobility. Physiological gut peristalsis and anatomical variations related to bowel and bladder filling introduce both inter‐ and intrafractional deformations and displacements of the target volume.^1^, ^2^, ^3^ In particular, the clinical target volume (CTV) of prostate cancer can experience inter‐ and intrafractional anatomic variations during external beam therapy with higher positional variability of seminal vesicles.^4^, ^5^

Rectal gas is present at the majority fractions in prostate cancer patients.^6^, ^7^, ^8^, ^9^, ^10^ Volumes of rectal gas > 20 mL were observed in about 5%–30 % of dose fractions.^7^, ^8^, ^10^, ^11^ Rectal distension by rectal gas is associated with increased intra‐fractional motion ^12^, ^13^, ^14^ and also with an increased risk of biochemical relapse following conformal radiotherapy without soft‐tissue image guidance.^15^ Intra‐fractional motion can displace the peripheral zone of the prostate and the anterior rectal wall, especially when an air pocket that distended the rectum during the planning CT is no longer present, resulting in a reduced posterior shift of the rectal wall. A rectal balloon can be used to displace the posterior rectal wall away from the planning target volume (PTV). Whether a rectal balloon may reduce inter‐ and intrafractional motion is an effect which has been inconsistently observed.^16^, ^17^, ^18^, ^19^, ^20^ Up to now, there is no high‐grade evidence for a rectal balloon. However, some retrospective studies have reported a reduction in toxicity associated with the use of a rectal balloon.^21^ The rectal balloon is usually filled with air for photon radiation therapy.^16^, ^22^ In addition, gas pockets in the bowel can appear and move inter‐ and intrafractionally.^23^, ^24^

Using a CBCT‐based online adaptive radiation therapy (ART) system, for dose calculation, a synthetic CT (generated by deforming the planning CT onto the initial cone beam CT (CBCT) of the treatment fraction) or an advanced CBCT integrated into the therapy system, which offers CT number accuracy comparable to that of a diagnostic spiral CT, is used.^25^, ^26^ A linear Boltzmann transport equation solver (LBTE) as calculation algorithm is implemented for dose optimization and final dose calculation. The linear Boltzman ionizing particle transport equation describes the macroscopic transport of ionizing particles, photons and electrons. It is sensitive for a disequilibrium of electrons due to tissue mass and electron density inhomogeneities.^27^, ^28^ A similar precision of Monte Carlo and LBTE dose calculations was found using inhomogeneous phantoms.^27^, ^28^, ^29^, ^30^ A reduction of the high‐dose region within the rectal gas / balloon air and the planning target volume overlap (PTV∩Air) has been observed in prostate cancer patients treated with IMRT, VMAT, or helical tomotherapy, based on Monte Carlo or LBTE dose calculations and validated through phantom measurements.^31^, ^32^, ^33^, ^34^, ^35^ This effect is attributed to the higher electron mobility within the air cavity, which leads to greater outward than inward scatter at the boundaries of small photon fields.^35^, ^36^, ^37^, ^38^ The optimizer than counteracts this decrease by increasing the fluence in the PTV∩Air to achieve the goals of dose homogeneity within the PTV. This phenomenon is described for so called class C optimizers based on LBTE or Monte Carle dose calculations.^39^, ^40^ Due to this optimizer sensitivity to inter‐ and intrafractional anatomic variations, the dose in the anterior rectal wall can increase, if it moves posteriorly and displaces the air at these positions in the PTV. This phenomenon has been observed also in the thoracic region analyzing inter‐ and intra‐fractional motion of the bronchial tree and the trachea within LBTE‐optimized lung cancer treatment plans.^39^

Though, anatomical deformations and density changes, are frequently observed during online ART, only a few studies addressed the issue of clinical robustness of ART workflows using an LBTE‐optimized dose calculation. In this study, the dosimetric robustness of LBTE‐optimized scheduled and adapted treatment plans for prostate cancer patients to inter‐ and intrafractional anatomical deformations shall be evaluated in the presence of intrarectal gas or an air‐filled endorectal balloon. D_max_, D_1_‐ and D_1cc_ changes in the rectal wall and in PTV∩Air‐structure overlap shall be evaluated as a function of graded water override of the air cavity and on inter‐ and intrafractional anatomical changes. Treatment plans were generated using a CBCT‐based adaptive radiation therapy platform. Hence, the primary aim was to identify the effects of intra‐ and interfractional anatomical changes on dosimetric characteristics, when using an online adaptive platform.

## 2 MATERIALS AND METHODS

### 2.1 Patient COHORTS and study concept

All patients of this study were treated with online adaptive radiotherapy on an ETHOS therapy system (Varian, A Siemens Healthineers Company, Darmstadt, Germany, V.1.0)). They gave informed consent to be included in prospective clinical registry approved by the local Ethics Committee before the start of treatment (18‐8364‐BO). This retrospective study had been approved by the local Ethics Committee (18‐8364‐BO). Treatments were performed between 01/12/2021 and 31/03/2023.

**FIGURE 1:**
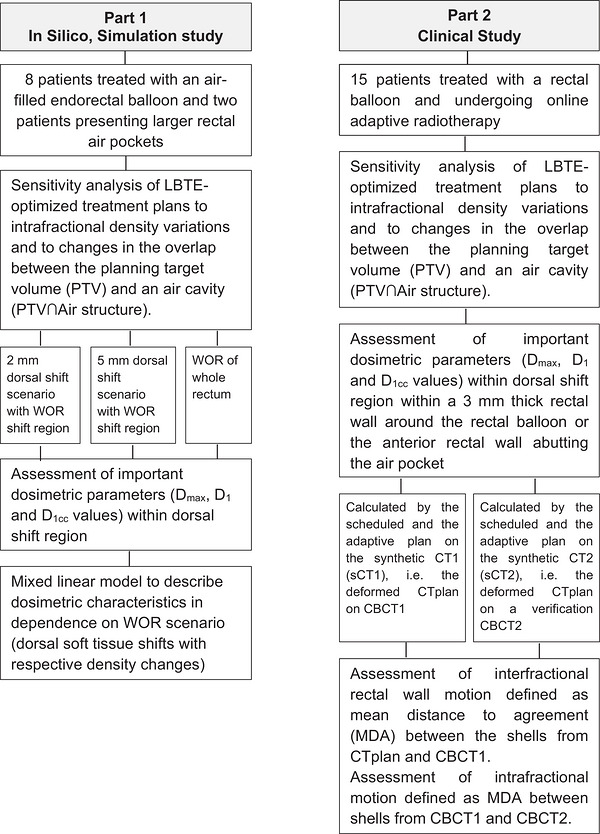
Figure 1 highlighting workflow diagram, part 1 and part 2 of study design.

This study was performed in two parts. The workflow diagram highlights the study design (Figure 1). The first part was an in‐silico simulation study, the second a clinical real‐life study. In the first part, a planning CT (CTplan) or a Hypersight cone‐beam CT (CBCT) (Varian, A Siemens Healthineers Company, Darmstadt, Germany) from ten patients with prostate cancer with intrarectal air was used. First, the effect of the width of PTV∩Air on the robustness of treatments plans, optimized with the Acuros XB dose calculation algorithm of the system (version AXB_16.1), against anatomical shifts of soft tissues into the air cavity was examined. Acuros XB dose calculation is used during iterative dose optimization and for final dose calculation. The default dose calculation grid is 2.5 mm. The Acuros XB algorithm as a linear Boltzmann transport equation (LBTE) solver, can predict doses at air‐water interfaces with comparable precision as Monte‐Carlo methods. The CBCT scans were reconstructed with advanced iterative algorithms and for improved scatter correction termed as iCBCT.^41^ Among the ten patients, eight patients were treated with a 75 mL rectal balloon (RectalPro75, QLRAD, Larnaca, Cyprus), while two patients presented with large gas pockets in the rectum. Patient 9 was treated without a rectal balloon and had the smallest air pocket in the rectum of a size of 18.3 mL, as captured on the cone‐beam CT during the course of treatment.

In the second part, we analyzed 101 dose fractions from 15 prostate cancer patients who underwent online adaptive radiotherapy ART on the ETHOS system using a rectal balloon. Eleven of them received definitive radiotherapy and 4 post‐prostatectomy salvage radiotherapy following biochemical recurrence. Maximum doses (D_max_), D_1_ (minimum dose in structure's 1% hottest voxel), and D1cc (minimum dose in structures hottest 1 cubic centimeter) within a 3 mm thick rectal wall around the rectal balloon or the anterior rectal wall abutting the air pocket were recorded, as calculated by the scheduled and the adaptive plan on the synthetic CT1 (sCT1), i.e. the deformed CT_plan_ on CBCT1 and by the adaptive plan on the synthetic CT2 (sCT2). The latter is the deformation of CT_plan_ on a verification CBCT2 acquired after calculation of the adaptive dose distribution during the online adaptive procedure just before the start of dose delivery. At this stage, image guidance can be performed to optimally align the adaptive plan with the anatomy on CBCT2. In addition, the geometric changes of the anterior surface of the rectal balloon between CT_plan_, CBCT1 and CBCT2 were determined. All radiation doses reported, were normalized by the prescribed dose.

### 2.2 Target volume expansions

**FIGURE 2:**
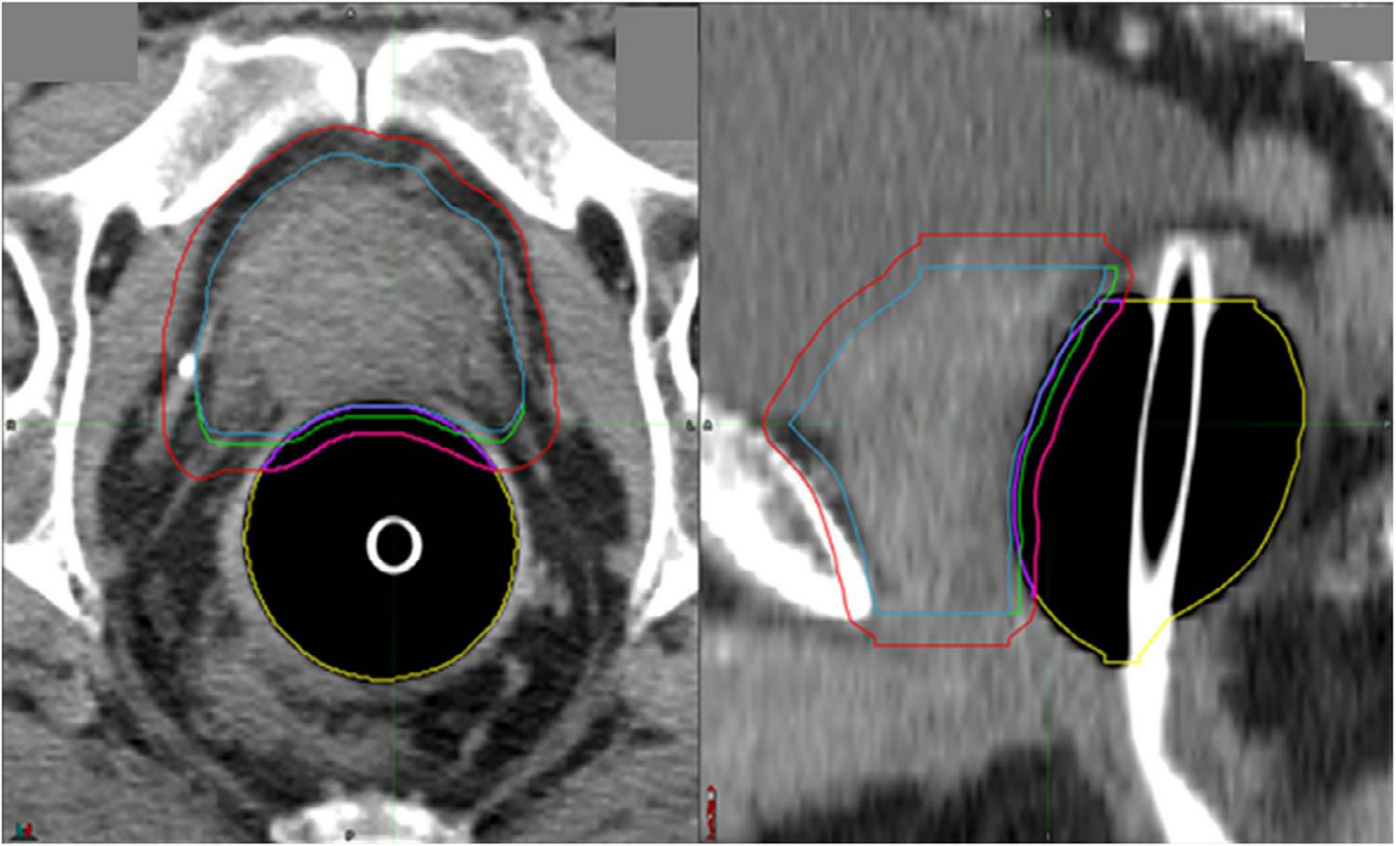
For the first part of this study, an isotropic 5 mm planning target volume (PTV, red contour) round the clinical target volume (CTV, inner blue contour) was used. Shift margins of 2 mm (green contour) and 5 mm in posterior direction from the CTV into the rectum were used for graded water override. The overlap of the 2 mm or 5 mm shift margin with the rectal balloon or air cavity, the PTV∩Air‐structure, was the resulting shift volume (magenta structure for 5 mm shift margin), that was used to simulate the posterior shift of soft tissue into the air cavity by water override (WOR). Left (A) axial CT view, right (B) sagittal CT view.

A uniform 5 mm PTV margin was used in the clinic for prostate cancer patients with a rectal balloon and for the simulation study evaluating graded WOR. Shift regions were constructed within the PTV by stepwise posterior expansion of the CTV into the rectum in 2 and 5 mm steps (Figure 2). Additional data on dorsal expansions resembling posterior shifts of more than 5 mm in 10 mm, 15 and 20 mm steps are given in the supplement (Figures S1,S2). The overlap of that expansion with the rectal balloon or air cavity, PTV∩Air‐structure, was the shift region that was used to simulate the posterior shift of soft tissue into the air cavity by water override (WOR). In addition to the PTV∩Air‐structure, a 3 mm thick rectal wall was contoured by a 3 mm expansion of the rectal wall of the rectal balloon or air cavity remaining after WOR. In contrast to the PTV∩Air‐structure that did not change with WOR, the rectal wall was shifted with the WOR of the shift region.

### 2.3 Treatment planning and dose recalculation

The dosimetric plan robustness refers to the sensitivity of a treatment plan's dose distribution to small changes in patient anatomy. The primary aim was to identify the effects of intra‐ and interfractional anatomical changes on dosimetric characteristics in the dose region within the rectal wall or the PTV∩Air‐cavity overlap. For the first part of this study, treatment plans were optimized on a 5 mm PTV that was used in the clinic for patients with a rectal balloon with the LBTE of the system. All adaptive and scheduled plan optimizations were performed using a predefined goal list. Translation of the goal list into the objective function was automatically carried out by the implemented Intelligent Optimization Engine (IOE). Target coverage of the clinical target volume (CTV) was prioritized with constraints requiring D_98%_ or D_99%_ ≥ 98%–99% at priority 1 (P1). For the planning target volume (PTV), coverage was generally prescribed as D_95%_ or D_96%_ ≥ 95%–96% (P1), while upper dose limits were set with D_1_ ≤ 107–110% (P1). Organs at risk (OAR) were constrained by a combination of dose–volume and mean dose objectives, with the bladder and rectum receiving the most detailed specifications. These included low‐ and intermediate‐dose volume limits as well as mean dose restrictions, with priorities assigned at P2–P3. The plans were subsequently exported to the treatment planning system Eclipse (Varian Medical Systems, Palo Alto, CA Version 16.00.00) for further dosimetric evaluation. Dose recalculation was performed using the same clinical dose calculation grid of 2.5 mm as utilized by default. To analyses the effects of anatomical changes of the position of the anterior rectal wall on dosimetric parameters, the dose distributions within WOR of the posterior shift regions of a thickness of 2 and 5 mm were calculated from the existing plans. In a further scenario the entire rectal balloon or air cavity was overridden with water density. In addition to the LBTE algorithm plans were recalculated by the Anisotropic Analytical Algorithm (AAA, Version AAA_16.1). Dosimetric characteristics were evaluated in the PTV∩Air‐structure and in the 3 mm thick rectal wall abutting the rectal balloon or air pocket. All dosimetric characteristics were normalized by the prescribed dose.

### 2.4 Rectal wall variability in adaptive prostate patients

For the second part of this study, D_max_, D_1_, and D_1cc_ values from the clinically applied plan were calculated for the 3 mm thick rectal wall on the synthetic CT1 (sCT1, derived from CBCT1) and the synthetic CT2 (sCT2, derived from CBCT2) for the respective dose fraction. Velocity deformation algorithm from the system was sensitive to the rectal gas. The deviations of the positions of the anterior surface of the rectal balloon copied from CBCT2 to sCT2 were minor in comparison to intrafractional anterior rectal balloon surface variation. Intrafractional rectal wall motion (from CBCT1 to CBCT2) and interfractional motion (from CT_plan_ to CBCT1 of the corresponding dose fraction) were assessed separately. This was done by expanding the CTV 8 mm posteriorly and quantifying its overlap with a 1 mm‐thick outer shell generated from the rectal balloon surface on each respective CT study. This shell was trimmed laterally and in superior/inferior direction so that it did not extent the x or z DICOM coordinates of the CTV center by more than ± 1 cm. To avoid edge effects in lateral, superior and inferior direction, all three shells from CT_plan_, CBCT1, and CBCT2 were further trimmed in x and z direction to the intersection area of the shells in x and z direction per dose fraction. This workflow was programmed in MIM (MIM Software Inc., Cleveland, OH, USA, version 7.3.2). The mean distance to agreement (MDA) between pairs of shells was computed to quantify displacement. Interfractional rectal wall motion was defined as MDA between the shells from CT_plan_ and CBCT1, whereas intrafractional motion was defined as MDA between shells from CBCT1 and CBCT2.

### 2.5 Statistics

Statistical analysis was performed using SAS software version 9.4, SAS/STAT15.1 (SAS‐Institute, Cary, NC). A mixed random and fixed effect model was built to describe D_max_, D_1_, and D_1cc_ characteristics within the rectal wall and the PTV∩Air‐structure by the WOR scenario as a fixed effect (Proc mixed, SAS). Furthermore, random intercepts dependent on the respective patient and the WOR scenario were introduced.

## 3 RESULTS

### 3.1 First part: Density variations and robustness of lbte‐optimized plans in the presence of air

In the first part of this study, the impact of density variations within the rectal gas cavity on the stability of LBTE‐optimized plans was investigated. D_max_, D_1_, and D_1cc_ values within the overlap volume (PTV∩Air) of the PTV with the rectum balloon or the intrarectal air cavity abutting the rectal wall were determined for plans with and without WOR within the posterior shift regions of 2 and 5 mm. Additionally, the respective dosimetric parameters were examined in the whole rectal air cavity or the whole rectal balloon. Plans optimized without WOR were rather homogeneous with average D_max_ values in the PTV with rectal balloon or air cavity overlap of 105.0% ± 1.2%. There was a close correlation between the D_max_, D_1_, and D_1cc_ values in the PTV with rectal air overlap regions characterized by Pearson correlation coefficients of 0.84 (95% CI: 0.46 – 0.96) between D_max_ and D_1_, 0.88 (95% CI: 0.56–0.97) between D_max_ and D_1cc_, and 0.74 (95% CI: 0.22 – 0.94) between D_1_ and D_1cc_.

Intrafractional anatomic shifts were simulated by expanding the prostate CTV in posterior direction by water‐override shift margins of 2, and 5 mm within the fixed PTV margin of 5 mm. Figures 3a, 3b show the D_1_ and D_1cc_ values within the original overlap region of PTV with the rectal balloon or the air pocket at the anterior rectal wall in dependence of the WOR. The D_1_ values increased with increasing shift margin with WOR of the whole intrarectal air cavity. Analysis with a linear mixed model showed a D_1_ increase estimates of 2.2% ± 0.7% and 5.6% ± 0.7%, for shift margins with WOR of 2 and 5 mm, and of 10.1% ± 1.4% for WOR of the whole rectal balloon (*p* < 0.0001, *F*‐test for the effect overall of WOR to the different extents). Similar increases were seen for the D_1cc_ and D_max_ values in the PTV with rectal balloon or air cavity overlap (Figure 2b). The D_1cc_ increase estimates were of 2.2% ± 0.7% and 5.5% ± 0.7%, for shift margins with WOR of 2 and 5 mm, and of 8.7% ± 1.0% for WOR of the whole rectal balloon (*p* < 0.0001, *F*‐test for the effect overall of WOR to the different extents). Similar D_max_ increase estimates were observed with 2.3% ± 0.7% and 5.8% ± 0.8%, for shift margins with WOR of 2 and 5 mm, and of 10.4% ± 1.5% for WOR of the whole rectal balloon.

**FIGURE 3A:**
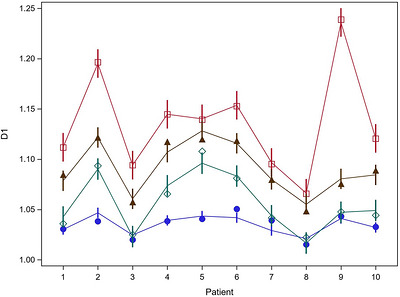
Observed D1 values within the overlap between the PTV of 5 mm and the rectal balloon or air pocket for the 10 patients, calculated on CTplan without water override (WOR) (blue circles), with 2 mm shift margin WOR (green diamonds), 5 mm shift margin WOR (brown triangles) and WOR of the whole intrarectal air cavity (red squares). In addition, predicted values by a linear mixed model were connected by a series line plot for the different WOR extents. 95% confidence intervals were also given for the predicted values as vertical bars. There was a significant dependence of D1 on the extent of WOR (p < 0.0001, F‐test). D1 increased with the extent of WOR by 2.2 ± 0.7%, 5.6 ± 0.7% on average for 2 mm, 5 mm shift margin WOR and by 10.1 ± 1.4% for WOR of the whole rectal air cavity.

**FIGURE 3B:**
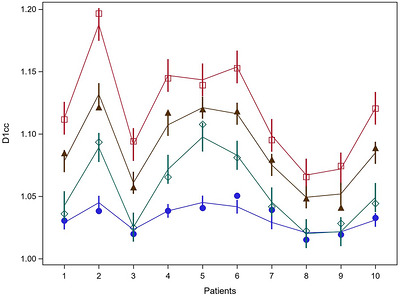
Observed D1cc values within the overlap between the PTV of 5 mm and the rectal balloon or air pocket for the 10 patients. For further descriptions, see Figure 3a.

The dosimetric characteristics were also analyzed in the rectal wall, adjacent to the rectal balloon or rectal air cavity. The rectal wall extended from the shift region to rectal air cavity interface 3 mm outward. For the scenario with WOR of the whole rectal air cavity, the rectal wall was kept the same as for the scenario without WOR. Figure 4a shows the D_max_ values in the rectal wall calculated for the scenarios with and without WOR of the whole rectal air cavity and with WOR of the 2 and 5 mm shift regions. Again, a significant effect of the extent of WOR was found by the linear mixed model. From the D_max_ estimate without WOR of 1.07 ± 0.06, D_max_ values increased by 1.7% ± 0.7% and 2.0% ± 0.7% of the prescribed dose for shift regions of 2 and 5 mm with WOR and by 6.1% ± 1.0% for WOR of the whole rectal air cavity (*p* < 0.0001, *F*‐Test indicating heterogeneity between the 4 scenarios with or without WOR to different extent).

**FIGURE 4A:**
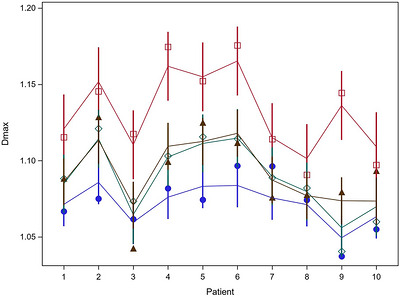
Observed Dmax values (a) within the rectal wall for the 10 patients, calculated on CTplan without water override (WOR) (blue circles), with 2 mm shift margin WOR (green diamonds), 5 mm shift margin WOR (brown triangles) and WOR of the whole intrarectal air cavity (red squares). For addition descriptions, see Figure 3a.

A similar dependence was observed for D_1_ and D_1cc_. From the D_1_ estimate without WOR of 1.06 ± 0.05, D_1_ values increased on average by 1.3% ± 0.7% and 1.7% ± 0.8% of the prescribed dose for shift regions of 2 and 5 mm with WOR and by 5.4% ± 0.8% for WOR of the whole rectal air cavity (*p* < 0.0001, *F*‐Test indicating heterogeneity between the 4 scenarios with or without WOR to different extent). The size of these effects varied from patient to patient (Figure 4b). For D_1cc_ of the rectal wall, the increase was 1.4% ± 0.6% and 1.7% ± 0.9% for shift regions of 2 and 5 mm with WOR and 5.0% ± 0.5% for WOR of the whole rectal air cavity (*p* < 0.0001, *F*‐Test for the overall WOR effect).

**FIGURE 4B:**
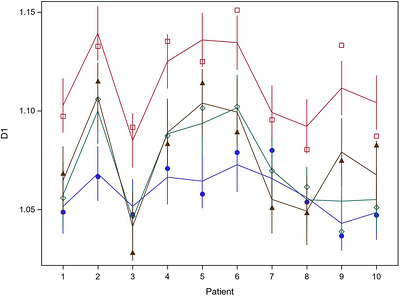
Observed D1 values (b) within the rectal wall for the 10 patients, calculated on CTplan without water override (WOR) (blue circles), with 2 mm shift margin WOR (green diamonds), 5 mm shift margin WOR (brown triangles) and WOR of the whole intrarectal air cavity (red squares). For addition descriptions, see Figure 3a.

**FIGURE 5:**
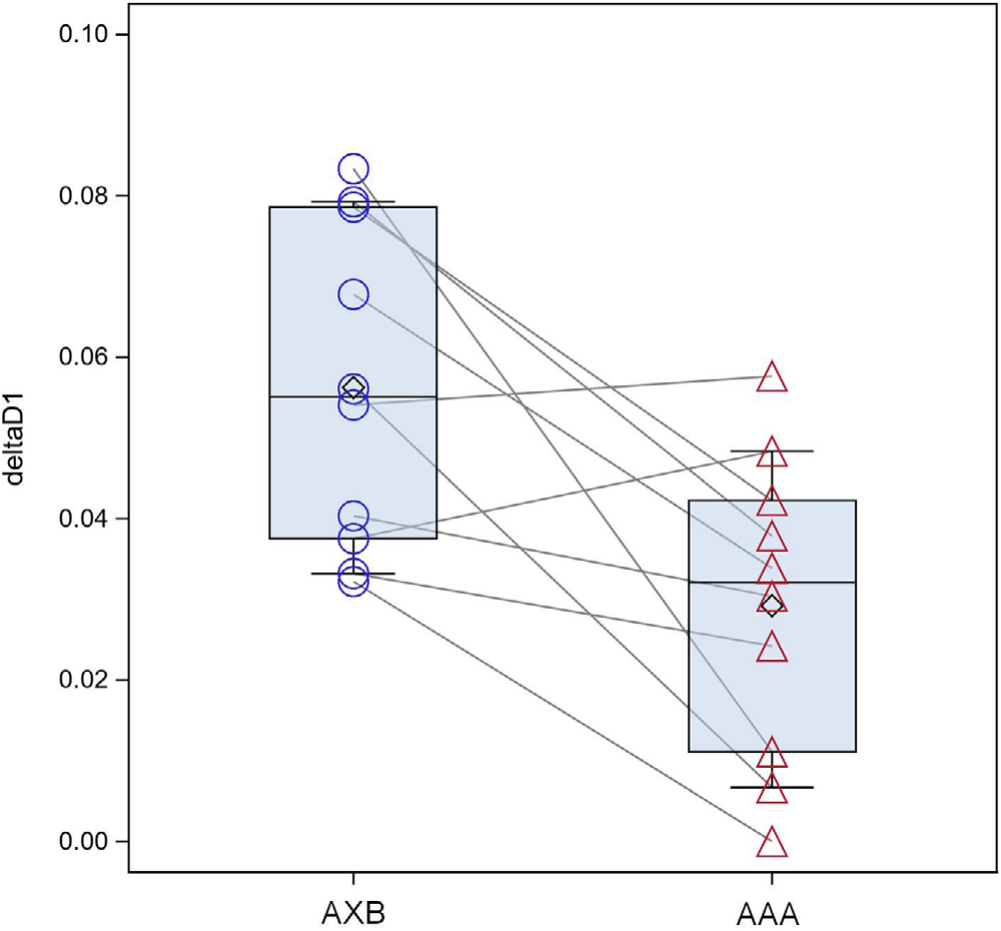
Box plots of the deltaD1 values (difference of the D1 calculated with minus without WOR) within the overlap between the PTV of 5 mm and the rectal balloon or air pocket in the 5 mm shift region. Dose calculations were performed with the AXB (LBTE‐algorithm) and AAA (CAA‐)algorithm. The height of the boxes indicates the interquartile intervals, the central horizontal line in the box the median. The respective values from the same dose fraction were connected by a grey drawn line. The whiskers extent from the 10th to the 90th percentile. The mean is indicated by a black diamond within the box.

The sensitivity of LBTE‐optimized and calculated plans was compared with the same plans recalculated with a contemporary convolution algorithm (CCA), the analytic anisotropic algorithm (AAA). Delta D_1_ values (difference of D_1_ values calculated on scenarios with minus without WOR in the 5 mm shift region) within the PTV with rectal air cavity/balloon overlap are given in Figure 5 for dose calculations with the LBTE and CAA algorithms. There was a decreased sensitivity of the CAA algorithm to WOR in comparison to LBTE (*p* = 0.0039, signed rank test). The CAA algorithms detected on average 56% ± 13% of the WOR effect found by LBTE. Similar observations were made for deltaD_1cc_. The CAA algorithms detected on average 43% ± 12% of the WOR effect found by LBTE at the D_1cc_ end point (*p *= 0.0020, signed rank test).

### 3.2 Second part: Density variations and plan robustness in the clinical setting of patients with prostate cancer undergoing adaptive radiation therapy

**FIGURE 6:**
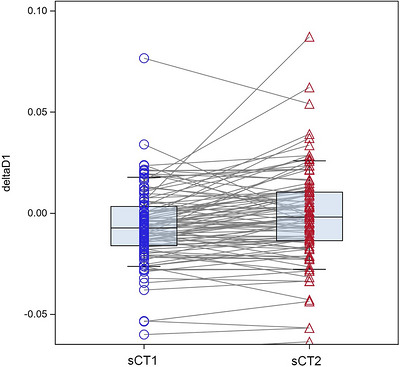
Box plots of the deltaD1 values in the rectal wall on sCT1 or sCT2 from 101 dose fractions. The delta D1 values are the difference of the D1 values in the rectal wall in the respective synthetic CT minus the D1 value in the planning CT. The height of the box indicates the interquartile intervals, the central horizontal line in the box the median. The respective values from the same dose fractions were connected by grey drawn line. The whiskers extent from the 10th to the 90th percentile.

In the second part of the study, we compared the intrafractional robustness of the D_max_, D_1_ and D_1cc_ values within the 3 mm thick rectal wall for patients treated with online adaptive radiotherapy and a rectal balloon. One hundred and one dose fractions from 15 patients were analyzed. Seven fractions were applied with the scheduled and 94 with the adaptive plan. Visual inspection showed a good agreement of the anterior surface of the rectal balloon between the synthetic CT and the respective CBCT. The time interval between CBCT1 and CBCT2 was on average 16 min. Figure 6 shows the deltaD_max_ values in the rectal wall calculated on the sCT1 and sCT2. deltaD_max_ values represent the difference between the D_max_ values in the rectal wall on the respective synthetic CT minus the D_max_ value on CT_plan_. The median deltaD_max_ value in the rectal wall for sCT1 was 0.0012 (90% CI: −0.0486–0.0280) and for sCT2 0.0043 (90% CI: −0.0381–0.0511). Therefore, no relevant systematic increases in the D_max_ values in the rectal wall were observed by shifts of the rectal wall from CT_plan_ to sCT1 or sCT2. The same holds for D_1_ and D_1cc_ (for D_1_, see Figure 5). The median deltaD_1_ value in the rectal wall for sCT1 was −0.0073 (90% CI: −0.0344–0.0210) and for sCT2 −0.0019 (90% CI: −0.0336–0.0337). The median deltaD_1cc_ value in the rectal wall for sCT1 was −0.0015 (90% CI: −0.0321–0.0194) and for sCT2 −0.0015 (90% CI: −0.0364–0.0292).

**FIGURE 7:**
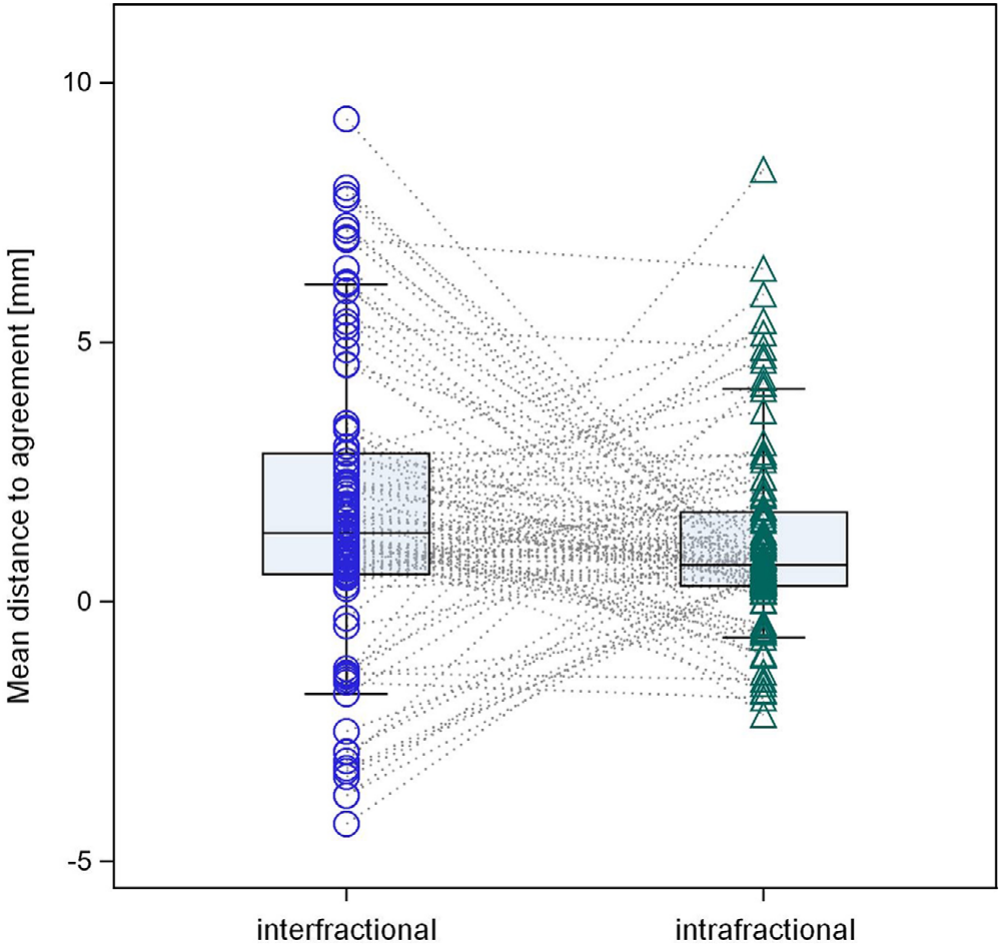
Box plots of the interfractional and intrafractional deviations of the anterior balloon surface, as measured by the mean distance to agreement (MDA). Positive shifts were in posterior direction, negative shifts in the anterior direction.

For analysis of inter‐ and intrafractional deviations of the anterior surface of the rectal balloon, the MDA between the anterior surfaces of the balloon on CT_plan_ and CBCT1 as well as between CBCT2 and CBCT1 were calculated. All shifts in posterior direction were labelled with a positive and in anterior direction with a negative sign. A minority of 19.8% of the interfractional and of 16.8% of the intrafractional shifts were in anterior direction. Figure 7 shows vertical box plots of the inter‐ and intrafractional MDA. The median interfractional MDA between the anterior wall contours of the rectal balloon was 1.3 mm, the median intrafractional MDA was 0.7 mm, with a nonsignificant difference between inter‐ and intrafractional MDA from the same dose fraction (*p* = 0.0616, signed rank test). The 90^th^ percentile of the interfractional and intrafractional MDA was 6.1 mm and 4.1 mm, respectively. At a PTV margin equal to MDA, 95% and 98% of the CTV was covered by the PTV in 100% and 83% of dose fractions.

## 4 DISCUSSION

The results of this study reveal that robust treatment plans for online ART can be achieved through dose optimization with the LBTE algorithm, when confronted with up to a 5 mm overlap with rectal air. Density variations in the overlap region (up to 5 mm), caused by anatomical differences, have only a moderate effect on D_max_, D_1_ and D_1cc_ in the rectal wall. In the present study, we observed an average D_1cc_ dose increase of 1.7% in the rectal wall due to a backward shift of 5 mm of the rectal wall displacing the rectal balloon. However, dose increases resulting from the water override method (WOR) may become even larger when the overlap exceeds 5 mm. A larger average D_1cc_ increase of 5% of the prescribed dose was observed, when the entire air cavity was replaced using the WOR method. The effects of more than 5 mm PTV∩Air overlap are reported in the supplement. Fortunately, typical PTV margins adequate for definitive radiation therapy of prostate cancer are 2 mm – 5 mm ^42^, ^43^, ^44^, ^45^, ^46^ and 3 mm – 8 mm for salvage radiotherapy.^47^, ^48^ The WOR approach allowed for the estimation of dose increases to the anterior rectal wall resulting from a posterior shift into the air‐filled rectal lumen. Such conditions may occur naturally due to intrarectal gas or are artificially introduced by a rectal balloon during plan optimization. The observed D_max_, D_1_ and D_1cc_ increases in the rectal wall due to posterior shifts up to 5 mm during online adaptation remained below 5.5% at the 95^th^ percentile in the present study, which is considered clinically insignificant. A similar observation for the D_max_ increase was made in the central bronchial wall using adaptive lung cancer treatment plans.^39^ Plan instability can largely be increased by density water overwriting the gas within the rectum during plan optimization as found in the present study and in the study by Soukup et al.^40^

Dose optimizers using Monte Carlo or LBTE algorithms effectively mitigate the dose buildup effect at the gas–rectal wall interface, which is typically observed with a homogeneous photon fluence field due to the longer electron range in air. This build‐up effect causes a rapid dose increase within the first 0.1 to 2 mm of tissue anterior to the air cavity created by a rectal balloon. Beyond 2 mm depth into the rectal wall, the dose reaches over 95 % of the prescribed value, as confirmed by phantom measurements.^33^, ^34^, ^49^ Within the balloon itself, the dose increases by more than 10% when a water override is applied compared to an air‐filled balloon, as demonstrated both in this study and in previous research.^34^

Applying online adaptive radiotherapy with MR‐linacs using an air‐filled endorectal balloon or a gas‐filled rectum, the problem of electron return effect rises, leading to a loop back of the electrons entering the air cavity to the rectal wall in the B‐field of the MRI.^35^, ^50^ However, reducing isotropic PTV margins from 4 mm with CT‐based image guidance to 2 mm with MR‐image guidance can reduce rectal toxicity of ultra‐hypofractionated prostate radiotherapy.^44^

The present study found that an endo‐rectal balloon did not lead to a significant reduction of intrafractional motion of the CTV in comparison to interfractional motion. CTV coverage in this study was achieved using 4.1 mm PTV margins in posterior direction defined by the 90^th^ percentile of the mean distance to agreement (MDA) between the anterior surface of the rectal balloon behind the prostate on pretreatment CBCT1 and verification CBCT2 of the same fraction. This CTV coverage was comparable to the CTV coverage obtained in the study by Byrne et al with a 5 mm PTV margin around the prostate in 90% of fractions.^45^ Despite the use of an endorectal balloon, rectal gas and stool remain significant contributors to intrafractional motion.^13^ While other studies have shown that endorectal balloons can reduce intrafractional motion compared to no balloon during definitive radiotherapy, motion tends to increase with treatment duration.^51^ In our practice, rectal balloons are used for rectal wall‐infiltrating tumors or for lymph node metastases located within 5 mm of the rectal wall. Primary goal of using rectal balloons is sparing the contralateral rectal wall. Similarly, the Tübingen group used endorectal ultrasound gel to separate the rectal wall during MR‐linac‐based dose escalation for rectal cancers.^52^ Deformable image registration is commonly used for structure propagation and dose accumulation during online adaptive radiotherapy. However, the appearance or disappearance of air pockets can violate the fundamental assumption of deformable registration—that a consistent correspondence exists between the images being aligned. To address this, several image modification techniques have been proposed to create or remove air pockets in one of the images to improve correspondence.^53^, ^54^ These methods, however, are not currently implemented in the deformable registration algorithms used for online adaptive radiotherapy. Despite these limitations, the resulting mapping errors have generally led to only minor reductions in the minimum PTV dose.^23^ It is also known from previous studies in bladder cancer that anatomical deformations can increase over the course of a treatment session.^16^ Similar findings have been observed in salvage radiotherapy, where the CTV may deform in a manner comparable to bladder cancer cases. Other studies have reported a progressive drift in prostate position during treatment, consistent with a random walk pattern over time.^55^, ^56^, ^57^ In future, multi‐institutional or larger datasets are important to further explore the issue of anatomical changes and its potential relevance in the clinical routine.

In summary, when the overlap between the PTV and rectal gas or balloon air does not exceed 5 mm, treatment plans using dose calculation with a LBTE algorithm as a representative for type C algorithms show dosimetric robustness to posterior soft‐tissue shifts of up to 5 mm. PTV∩Air‐overlaps of 5 mm led to LBTE‐optimized treatment plans, that showed only minor sensitivities to anatomic soft tissue shifts of 2–5 mm in posterior direction with respect to dose homogeneity. During online adaptive radiotherapy, the presence of rectal air pockets or an air‐filled rectal balloon did not result in clinically relevant dose increases, provided that intrafractional motion–induced soft tissue shifts remained within 5 mm in the posterior direction. Under these conditions, dose homogeneity was not found to be compromised by anatomic changes to a clinically relevant extent. However, PTV∩Air larger than 5 mm led to LBTE‐optimized treatment plans that are sensitive to anatomic soft tissue shifts larger than 5 mm in posterior direction with respect to dose homogeneity. These findings highlight the importance of minimizing adaptation time during online adaptive radiotherapy to allow for the use of the smallest possible PTV margins.

## AUTHOR CONTRIBUTIONS

The authors have nothing to report.

## FUNDING INFORMATION

This research did not receive any specific grant from funding agencies in the public, commercial, or not‐for‐profit sectors.

## CONFLICT OF INTEREST STATEMENT

All authors declare that there is no conflict of interest.

## ETHICAL APPROVAL

The prospective clinical registry (18‐8364‐BO) and the retrospective study analysis had been approved by the local Ethics Committee (18‐8364‐BO).

## Supporting information



Supporting Information
