# Supplementary material for: Effects of air on the dosimetric robustness of treatment plans for prostate cancer in the presence of intrafractional, anatomical changes during online adaptive radiotherapy
Source: J Appl Clin Med Phys. 2026 Jul 7;27(7):e70675. doi: 10.1002/acm2.70675 (PMC13341946; doi:10.1002/acm2.70675)
Supplement: Supplementary file 1 — Supporting Information [file ACM2-27-e70675-s001.zip › 2025-09007-s01.docx]

**Effects of air on the dosimetric robustness of treatment plans for prostate cancer in the presence of intrafractional, anatomical changes during online adaptive radiotherapy**

Nika Guberina ^a,b,#^; Aymane Khouya ^a^; Alina Santiago Garcia ^a^; Christoph Pöttgen ^a^; Thomas Gauler ^a^; Gerrit Fischedick ^a^; Christopher Darr ^c^; Toke Ringbaek ^a^; Maja Guberina ^a,b^; Martin Stuschke ^a,b^

^a^ Department of Radiotherapy, West German Cancer Center, University Hospital Essen, Germany

^b^ German Cancer Consortium (DKTK), Partner Site University Hospital Essen, Germany

^c^ Department of Urology, West German Cancer Center, University Hospital Essen, Germany

^#^ corresponding author

**Funding:**

This research did not receive any specific grant from funding agencies in the public, commercial, or not-for-profit sectors.

**Conflict of interest:**

All authors declare that there is no conflict of interest.

**Keywords:** adaptive radiation therapy, prostate cancer, dose inhomogeneity, plan robustness

**Word count manuscript:** ~4900 words

**Word count abstract**: 495 words

**Corresponding Author #**

| Name | Prof. Dr.med. Nika Guberina |
| --- | --- |
| Address | University Hospital Essen, Department of Radiotherapy, West German Cancer Center (WTZ), Hufelandstraße 55, 45147 Essen, Germany |
| Phone | +49 201 723 82282 |
| Fax | +49 201 723 5610 |
| E-mail | nika.guberina@uk-essen.de |
