# Supplementary material for: Effects of air on the dosimetric robustness of treatment plans for prostate cancer in the presence of intrafractional, anatomical changes during online adaptive radiotherapy
Source: J Appl Clin Med Phys. 2026 Jul 7;27(7):e70675. doi: 10.1002/acm2.70675 (PMC13341946; doi:10.1002/acm2.70675)
Supplement: Supplementary file 1 — Supporting Information [file ACM2-27-e70675-s001.zip › 2025-09007-sup-0002-SI_Figure-S01.docx]

**Supplement**

**Supplementary Figure 1**


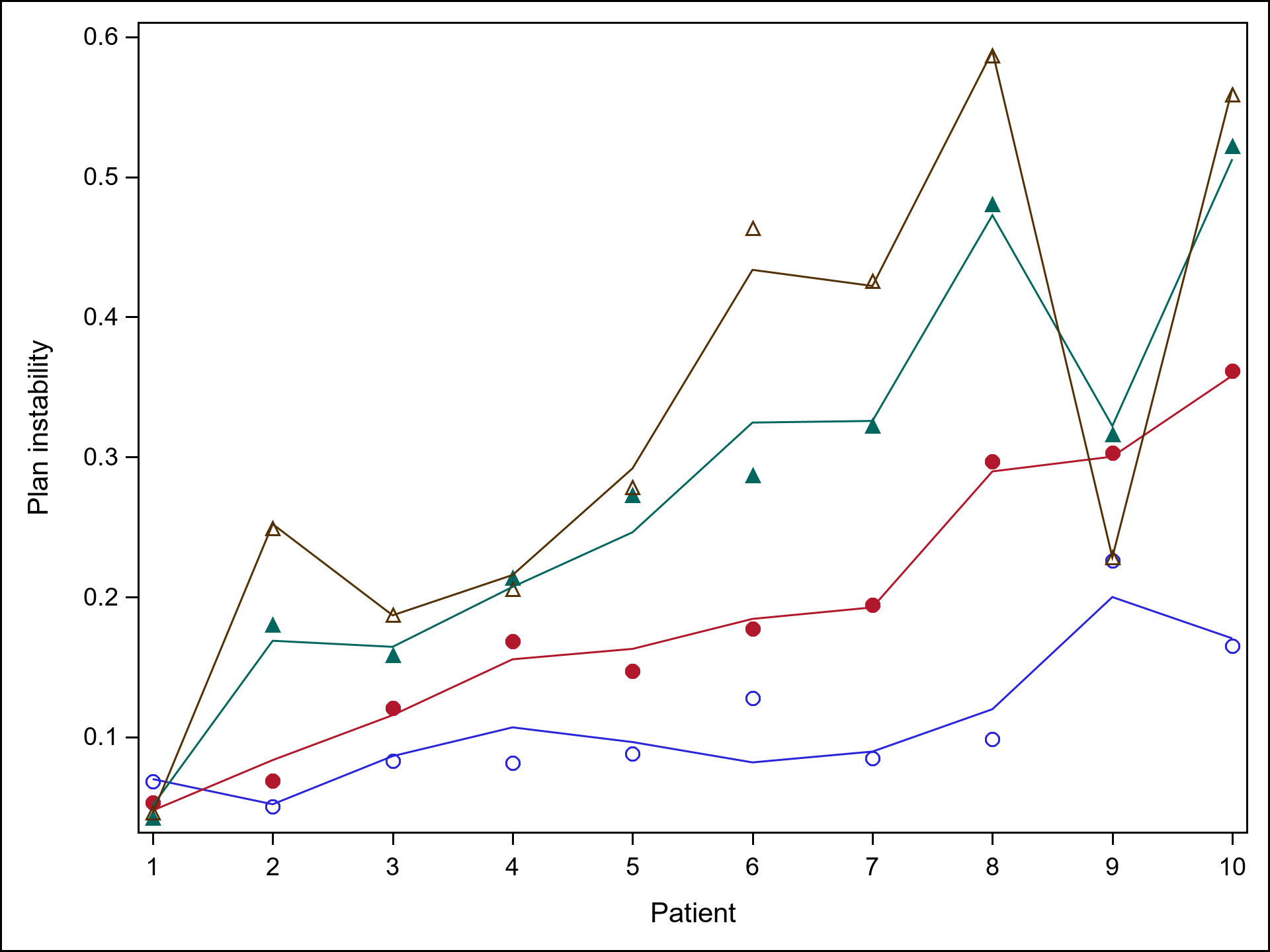


Supplementary Figure 1 shows a random slope and random intercept by patient model for the plan instability, determined as Delta Dmax within the whole rectal air cavity with and without water overwrite (WOR), in dependence on the posterior width of the PTV margin toward rectal air as a classification variable. Plan instability is defined as Dmax of the LBTE optimized plan with WOR of rectal air - Dmax of the LBTE optimized plan without WOR. Data are given with different colors in dependence on the posterior PTV margin. Blue, red, green and brown curves and data: posterior PTV margins of 5 mm, 10 mm, 15 mm, and 20 mm margins with respective rectal air lumen overlaps. Patients are ordered according to increasing plan instability at 10 mm margins. Predicted values for the different PTV margins, derived from a mixed model, are connected by line segments with random slopes and intercepts per patient. The effect of PTV margin width on plan instability was significant (p=0.0015, F-test).

**Supplementary Figure 2a**

**Supplementary Figure 2b**

Figure 2 a and b: Dmax (a) or Dmean (b) values normalized by the prescribed dose within the posterior shift region with water-override (WOR) in dependence of the plan instability parameter, and the posterior width of the posterior water override shift region. Data from 5 mm, 10mm, 15 mm and 20 mm shift regions are given with blue, red, grey and green symbols.
